# The S100A10–AnxA2 complex is associated with the exocytosis of hepatitis B virus in intrauterine infection

**DOI:** 10.1038/s41374-021-00681-8

**Published:** 2021-10-13

**Authors:** Xiaoxia Bai, Jinshi Ran, Xianlei Zhao, Yun Liang, Xiaohang Yang, Yongmei Xi

**Affiliations:** 1 The Women’s Hospital, Zhejiang University School of Medicine, No. 1 Xueshi Road, Shangcheng District, Hangzhou, Zhejiang 310001 China; 2 Institute of Genetics and Department of Human Genetics, Zhejiang University School of Medicine; Zhejiang Provincial Key Laboratory of Genetic & Developmental Disorders, No. 866, Yuhangtang Road, Hangzhou, Zhejiang 310058 China; 3 Joint Institute of Genetics and Genomic Medicine between Zhejiang University and University of Toronto, Zhejiang University, No. 866, Yuhangtang Road, Hangzhou, Zhejiang 310058 China

**Keywords:** Hepatitis B, Infection

## Abstract

Mother-to-child transmission (MTCT) is the major cause of chronic infection of hepatitis B virus (HBV) in patients. However, whether and how HBV crosses the placenta to cause infection in utero remains unclear. In this study, we investigate the mechanism as to how the HBV virions pass through layers of the trophoblast. Our data demonstrate the exocytosis of virions from the trophoblast after exposure to HBV where the endocytosed HBV virions co-localized with an S100A10/AnxA2 complex and LC3, an autophagosome membrane marker. Knockdown of either AnxA2 or S100A10 in trophoblast cells led to a reduction of the amount of exo-virus in Transwell assay. Immunohistochemistry also showed a high expression of AnxA2 and S100A10 in the placental tissue samples of HBV-infected mothers with congenital HBV-positive infants (HBV^+/+^). We conclude that in HBV intrauterine infection and mother-to-child transmission, a proportion of HBV hijacks autophagic protein secretion pathway and translocate across the trophoblast via S100A10/AnxA2 complex and multivesicular body (MVB)-mediated exocytosis. Our study provides a potential target for the interference of the mechanisms of HBV intrauterine infection and mother-to-child transmission.

## Abstract (web summary)

Mother-to-child transmission is the major cause of chronic hepatitis B virus (HBV) infection. This study shows that an unconventional protein secretion pathway that depends on autophagy may be hijacked by HBV to complete the process of intracellular transport. In HBV-infected trophoblasts, AnxA2-S100A10-mediated exocytosis may result in HBV intrauterine transmission.

## Introduction

China has one of the highest prevalences of HBV infections across the world, encompassing up to 10% of the entire population^1^. More than 50% of chronic patients have been found to have been infected through perinatal mother-to-child transmission. Those infected with HBV during childhood have a higher chance than the rest of the population of encountering diseases associated with liver injury in adulthood^2^. Even though a variety of measures have been taken to prevent mother-to-child transmission over the previous years, such as reducing viral load in pregnancy and the combination of active–passive immunization in newborns, 6–11% of such efforts have failed due to intrauterine transmission. This remains the main cause of the lingering high proportion of viral carriers^3,4^. As a consequence, it is of great significance to study the intrauterine transmission mechanism of HBV with the aim of reducing the rate of chronic HBV carriers and the incidence of HBV infection.

HBV invades the trophoblast before entering fetal blood circulation, a process in which the trophoblast acts as a critical component of the maternal-fetal blood barrier. Clinical studies have shown that HBV intrauterine transmission occurring through transplacental transmission could be hematogenous^5^ or occur by extracellular transfer^6,7^. HBV-infected maternal blood leakage into the infant circulation may have occurred in cases of placental abnormalities, local tissue lesions, or preterm labor^5^. The presence of HBV, including HBsAg and HBV DNA, in placental tissues, has been detected in 5 cases out of 6 infants with positive cord blood, though at relatively low levels^8^. The cellular transfer has been proposed as the main route for HBV intrauterine transmission^9^. HBV virions in maternal blood circulation enter the intervillous space where they infect trophoblasts and mesenchymal cells, get past villous capillary endothelial cells and then enter infant blood circulation^10^. Wang et al. detected 323 cases of newborns within 24 h of the detection of a serum HBV-DNA positive rate, in which they found that there was about 2% neonatal serum HBV-DNA > 100 IU/ml and high levels of HBV-DNA. This suggested that these fetuses had been infected with HBV in the uterus^11^. Other studies have also shown that HBV not only infects primary trophoblast cells but also duplicates itself and translocates across the placenta^12,13^. Testing of HBsAg, HBcAg, and HBV-DNA conducted upon placental tissues from 171 HBV-infected pregnant women showed that among different cell layers of the placenta, the decidua had the highest HBsAg, HBcAg, and HBV-DNA positivity, followed by trophoblasts, and then others^14^. These observations suggest that replicated HBV virions undergoing exocytosis from trophoblasts are a key component in HBV intrauterine infection. However, no mechanism of HBV exocytosis from trophoblasts has yet been reported.

We recently identified a high level of S100A family members in the serum samples of HBV^+/+^ infants, compared to that of HBV^+/−^ infants. Some of these S100A proteins (S100As) were also detected to be highly expressed in the placental tissue of samples obtained from HBV-infected patients^15^. Chen et al.^16^ reported that exogenous IFN-γ-induced autophagy in lung epithelial cells is involved in the extracellular secretion of cytoplasmic AnxA2 and that the relocation of AnxA2 from the autophagosome to amphisome requires Rab11. AnxA2 is a key player functioning in such biological processes in membrane transfer, endocytosis, and vesicle transportation^17,18^. AnxA2 is also a known participant in various pathological processes such as cancer development or bacterial or viral infection, these processes requiring Ca^2+^-dependent S100A10 accompaniment^19,20^. In such processes, activated S100 combines with AnxA2 and then promotes the assembly of microfilaments (MFs) and actin, leading to MF-dependent cytoskeleton rearrangement and facilitating vesicle exocytosis^21^. For instance, the S100A10/AnxA2 complex mediates the exocytosis of vWF in vascular endothelial cells^22^, BTV in BHK21 cells^23^, and HPV in human keratinocytes^24,25^. Taken together, we speculated that the intrauterine transmission of HBV might utilize a similar vesicle transport mechanism to achieve maternal and infant infections through S100A10/AnxA2-mediated exocytosis in placental cells.

Autophagy can selectively target intracellular pathogens^26^. It plays an important role in the presentation of extracellular antigens to MHC class II mollecules^27^. Some bacteria and viruses have developed strategies to inhibit or bypass autophagy to ensure their survival^28^. Viruses such as poliovirus, rhinovirus, mouse hepatitis, and SARS-CoV, even hijack components of the autophagy process and utilize them for their own replication^29,30^. In hepatic cells, HBV uses autophagosomes to promote the processes of DNA replication, assembly, maturation, and secretion^31^. The targeted knockdown of the autophagy proteins Atg5, Atg7, or Beclin1 also reduces HBV release^32^. In HBV-infected liver cells, Rab7 also plays a central role in regulating HBV transport and secretion^33^. Rab7 is involved in multiple endocytosis processes, including late endosomes/multivesicular body (MVB), autophagosomes, lysosomes, and other lysosome-related organelles^34^. The knockdown of Rab7 or the inhibition of lysosomal function led to increased intracellular accumulation and secretion of HBV. Rab7–mediated autophagy pathways protect cells against fatal acute HBV infection^35^. In addition, recent studies have reported that autophagy also plays an important role in protein trafficking and secretion, enabling leaderless cytosolic proteins to not enter the conventional endoplasmic reticulum (ER)-to-Golgi pathway, but instead be secreted from the cytosol via the autophagic pathway^36^. This suggests that in HBV-infected cells if no acute inflammation occurs, the Rab7-mediated autophagy pathway could play a similar role for HBV exocytosis.

In the present study, we screened several S100A proteins and Anx2 using immunohistochemical staining in the placental tissue samples obtained from HBV^+/+^ and HBV^+/−^ patients and HBV^−/−^ controls. The primary cultured human trophoblast cells, trophoblast cell lines Swan71 and BeWo were induced by human HBV-positive serum and showed both high expressions and colocalization of S100A10 and Anx2. Colocalizations of various combinations of AnxA2, HBV, or LC3 were also detected by immunofluorescent staining in the cells. In addition, our data also shows that Rab7 mediates HBV intracellular trafficking to the lysosomes. In short, the unconventional protein secretory pathway related to autophagy is likely to be utilized by HBV for intracellular trafficking and exocytosis under the conditions of HBV intrauterine infection. Our study provides a potential target for interfering with the mechanisms of such HBV intrauterine infections and related mother-to-child transmission.

## Methods and materials

### Subject details and ethics statement

The cases of full-term pregnant women who gave birth were selected from January 2014 to December 20015 in the Women’s Hospital of Zhejiang University. The human placenta was collected with permission immediately beyond delivery. Villi samples were obtained from the miscarriage of early pregnancy (4 weeks) with the patient’s consent at the Women’s Hospital, School of Medicine, Zhejiang University. This study was approved by the Women’s Hospital of Zhejiang University ethics committee (IRB-20200019-R) and informed consent was acquired from donors before sample collection.

### Virus and cells

HBV was acquired from HBV-infected patients, with ethical approval from the hospital ethics committee and informed consent from the patients. The trophoblast cell lines Swan71 and BeWo (from the Women’s Hospital of Zhejiang University) were cultured in DMEM/F12 medium supplemented with 15% fetal bovine serum at 37 °C with 5% CO_2_.

### Immunohistochemistry

In total, 30 placental tissues were obtained from healthy women (HBV^−/−^), 30 from HBV-infected women with uninfected infants (HBV^+/^^−^), and 30 from HBV-infected women with infected infants (HBV^+/+^). For immunohistochemical staining, placental samples were collected immediately after delivery. Tissues of 1 cm × 1 cm × 1 cm were taken at different sites of placentas to be fixed in 10% formalin, embedded in paraffin, and sliced to a thickness of 5 µm. The paraffin sections were subjected to conventional dewaxing and hydration, and the antigen was heat-repaired in a pH 6.0 buffer of sulfate. After treatment with 1% H_2_O_2_, primary antibodies were applied to sections according to the manufacture’s instruction. After being rinsed with TBST, the sections were incubated with horseradish peroxidase (HRP)-conjugated secondary antibodies at room temperature for 30 min. Then sections were incubated with the substrate solution containing 3, 3′-diaminobenzidine (DAB), counterstained with Meyer’s hematoxylin, and routinely dehydrated through graded ethanol and xylene. Images were taken using a Nikon DsRi2 and analyzed using ImageJ. Observation of brown spots in the cytoplasm was considered positive. The negative control used pre-immune rabbit IgG to carry out the above reactions.

### Transmission electron microscopy (TEM)

Swan71 cells were seeded onto a dish to reach 90% confluence prior to co-incubation with serum-containing HBV at MOI_100_ for 24 h. Then cells were trypsinized, washed with phosphate-buffered saline (PBS), and centrifuged to collect the pellets. The cells were first fixed with glutaraldehyde, then rinsed in PBS, and secondary fixation was then carried out with OsO_4_. Cells were dehydrated through graded ethanol prior to embedment with resin and then cut into fine sections of 50 nm. Then the particles were visualized using a transmission electron microscope.

### Quantitative real-time polymerase chain reaction (PCR)

To quantify the amount of HBV, HBV DNA was extracted from the patient’s sera or culture medium using a Viral DNA kit (Omegabiotek, D3892) according to the manufacturer’s instruction. qPCR was performed as described previously. Briefly, primers used for amplification were: GGGAGGAGATTAGGTTAA and GGCAAAAACGAGAGTAACTC. HBV 1.3-mer WT replicon (Addgene, plasmid#65459) was used as standards. Serially diluted standards were processed in parallel with samples to generate a standard curve for quantification.

### Transwell assay

Swan 71 was seeded on Transwell insert plates (Corning, 3491) with 5% CO_2_ at 37 °C for 24 h to obtain a compact monolayer, of which TEER was detected with Milicell ERS-2 (Milicell). TEER > 200 Ω/cm^2^ indicates a compact monolayer. Serum containing HBV was added to the upper or lower chamber to reach an MOI for 100 and co-incubated for 1, 2, 4, 8, 12, and 24 h. At the end of co-incubation, the lower or upper supernatant was collected and sent to RT-PCR for viral concentration to determine transcytosis efficiency.

### Isolation and purification of primary trophoblastic cells

Freshly removed villi were rinsed 3 times with a cold 0.9% saline buffer to remove remaining blood. Tissues were minced with scissors and blood vessels were carefully removed. Mincedtissues were then placed in a 15 ml centrifugation tube filled with 10 ml digestion solution (Collagenase I dissolved in D-PBS at a concentration of 5 mg/ml) and the tube was placed in a 37 °C water bath and manually shaken every 10 min until the solution turned turbid. Then the tube was removed from the water bath and tilted for 1 min to sediment any remaining tissues. The supernatant was carefully transferred into another tube to neutralize Collagenase I with a complete culture medium. The mixture containing disassociated cells was filtered through a cell strainer (40 µm, Falcon, 352340). To collect the primary placental cells, the filtered supernatant was centrifuged at 1000 rpm for 5 min. The supernatant was then discarded and the pellet resuspended in a culture medium containing penicillin–streptomycin (Yeason, P8926790), where the disassociated cells were cultured overnight. On the second day, cells were trypsinized, centrifuged, and seeded onto a dish to allow attachment for 20–30 min, during which fibroblasts were expected to adhere preferentially. The medium containing unattached cells was transferred to the second dish to allow the second round of attachment for 20–30 min to remove fibroblasts and other contaminating cells. Primary trophoblast cells were identified, and the purity of the isolated populations was assessed using immunostaining of CK-7 (a marker for trophoblasts).

### Immunofluorescent staining

Cells were co-incubated with serum-containing HBV at a MOI_100_ for 6, 12, 24, and 36 h. For colocalization studies, cells were washed with PBS three times and fixed with 4% paraformaldehyde-PBS for 10 min at room temperature, cells were then washed with PBS and permeabilized with 0.4% Triton-PBS for 15 min. The cells were then washed with PBS and blocked with 5% bovine serum albumin–PBS for 1 h at room temperature. Cells were stained with primary antibodies at 4 °C overnight followed by secondary antibodies at room temperature for 1 h. The nuclei were stained with DAPI (Solarbio, C0065) at room temperature for 10 min. Confocal images were taken using an Olympus FV1000. Uninfected cells were used as control.

### siRNA transfection

The siRNA oligos were purchased from Genepharma (Shanghai, China). According to the manufacturer’s protocol, AnxA2 siRNA, S100A10 siRNA, and negative control siRNA were transfected into Swan71 cells using Lipofectamine RNAi-MAX reagent (Invitrogen). Briefly, 4 × 104 cells were seeded into a 24-well plate and cultured to reach 50% confluence. RNA was added to each well to a final concentration of 40 nM and incubated for 24 h before further analysis. In the assays, the negative control siRNA was used as the control.

### Western blot

Western blot assays were used to detect the expression of AnxA2 and S100A10 in Swan71 cells. In the assays, cells infected with HBV at a MOI_100_ for 6, 12, 18, and 24 h were lysed with a RIPA buffer (Solarbio, R0010) supplemented with PMSF and protease inhibitor cocktail (Roche). Total protein content was determined using the BCA (Thermo Fisher, 23225). Total cell lysates were mixed with 5× loading buffer and heated at 95 °C for 5 min and then separated by sodium dodecyl sulfate-polyacrylamide gel electrophoresis. Proteins were transferred to polyvinylidene fluoride membranes. After blocking with 5% non-fat milk diluted with TBS-0.2% Triton, blots were incubated with primary antibodies at 4 °C overnight. After washing with TBS-0.2% Triton 3 times, blots were incubated with HRP-conjugated secondary antibodies for 1 h at room temperature. Blots were developed and images were taken with Clinx Chemiscope.

### Ca^2+^ signal detection

Cells were co-incubated with serum-containing HBV at a MOI100 for 6, 12, 24, and 36 h, and then were trypsinized, washed with PBS, and centrifuged at 1000 rpm for 5 min to remove adherent HBV particles. The precipitate was resuspended with PBS, and 100 µl of a homogenized mixture containing Swan71 cells were loaded onto glass slides, and time is given to air dry. For calcium fluorescence probing, Fluo-4 AM (Beyotime, S1060) was added to the slides according to the manufacturer’s instructions. Confocal images were taken with Olympus FV1000. Uninfected cells were used as control.

### Quantification and statistical analysis

Pearson’s colocalization coefficients were conducted using ImageJ software. Results from at least three independent experiments were presented as the mean ± SD. One-way analysis of variance (ANOVA) followed by Dunnett’s multiple comparisons test was used to determine significant differences. *P* < 0.05 was considered significant.

## Results

### S100A proteins were highly expressed in the placenta and the serum of HBV infectious infants

**Fig. 1:**
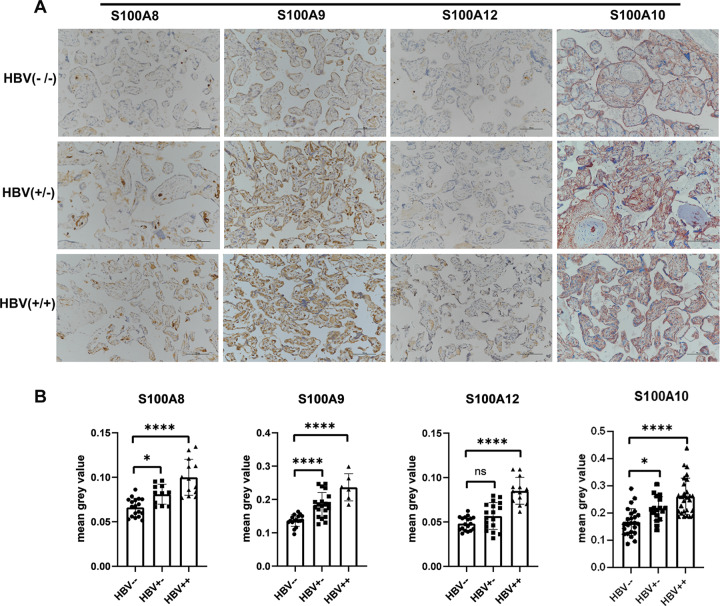
High expression of S100As in the placental tissues of patients with HBV infection. **A** Immunohistochemical staining of S100A8, A9, A12, and A10 in the placental tissues of infected patients (HBV^+/−^, HBV^+/+^) and the control (HBV^−/−^). **B** Gray analysis of immunohistochemical staining results show that S100A8, A9, A12, and A10 are highly expressed in HBV^+/+^ and HBV^+/^ placental tissues, but S100A12 did not show a significantly high expression in HBV^+/−^ placental tissues. Scale bars indicate 10 μm.

Having received consent, we collected placental tissue samples of 30 of each of HBV^+/+^, HBV^+/^^−^, and HBV^−/−^ clinical cases, 90 cases total. Sections were used for immunohistochemical staining to screen the expressions of the S100A protein family. We found that the expression of S100A10 was significantly increased in both HBV^+/+^ and HBV^+/−^, compared to the controls (Fig. 1A, B). In addition to this substantive difference in S100A10 expression, we also re-assed the expressions of S100A8, S100A9, and S100A12, which coincide with our previous results^15^. High expressions of S100A8, S100A9, and S100A12 were detected in the placental tissues of both HBV^+/+^ and HBV^+/−^, compared to that of HBV^−/−^ controls (Fig. 1A, B). As an exception, there was no significant difference in the expression of S100A12 between HBV^+/−^ and HBV^−/−^. S100A10 was also not detected to have a high expression in the HBV^+^ infant serum samples^15^, probably due to S100A10 being mainly distributed in the intracellular and plasma membrane and not secreted into the peripheral blood^37^. The above results, minus the exceptions, indicate that the expression levels of S100As in the placenta are positively correlated with HBV infection.

### Infection of HBV in human trophoblast cells and its transmission in a trophoblast organoid

**Fig. 2:**
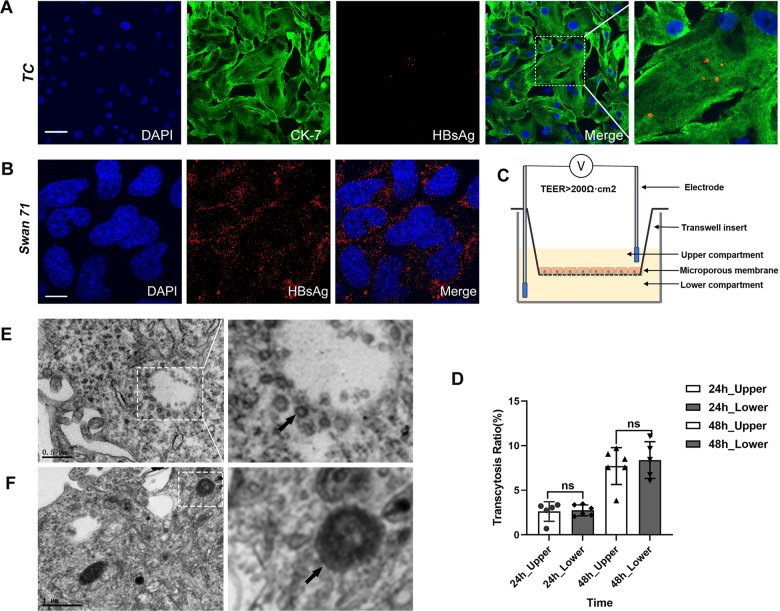
Infection of HBV in human trophoblast cells and its transmission in a trophoblast transwell model. **A** Immunofluorescent staining showing that HBV can infect human trophoblast cells (TC). Scale bars indicate 50 μm; **B** Immunofluorescence staining showing that HBV can infect Swan71 cells. **C** Transwell method to form dense monolayer Swan71 cells after 24 h of culture. The transmembrane resistance was greater than 200 Ω cm^2^. **D** Transwell assay shows that HBV can infect Swan71 trophoblast cells and complete exocytosis. There was no difference in the amount of cyto-virus infected from the top or bottom surfaces of the cells. **E**, **F** Transmission electron microscopy showing that virus particles and dense lysosomes were observed in Swan71 cells 48 h after HBV infection. Scale bars indicate 10 μm.

To obtain insight into the mechanisms of HBV intrauterine infection, we collected human villous tissue samples with permission. The trophoblast cells were isolated using a differential adhesion method, and cultured using a protocol described previously, with minor modification. After purification, the trophoblast cells were induced by human HBV+ serum (MOI 1:100) for 24 h and then mounted for immunofluorescent staining using an HBsAg-against sheep antibody. The signals of HBV infection and a marker of human trophoblast cells, cytokeratin-7 (CK-7), were observed under an Olympus confocal microscope (Fig. 2A). In primary human trophoblast cells, the efficiency of the HBV infection did not seem to be high. We then selected a human trophoblast cell line, Swan71, as a model system to perform the following experiments. Firstly, the Swan71 cells were cultured and exposed to HBV serum (MOI 1:100) for 24 h. Immunofluorescent staining showed that most of the Swan71 cells showed HBV infection (Fig. 2B). We then used the Transwell culture method to construct an in vitro trophoblast cell model to simulate the trophoblast cells as a monolayer in the placental tissue. When the trophoblast cells had formed dense monolayer cells in the Transwell chamber (Fig. 2C), the transendothelial electrical resistance (TEER) was greater than 200 Ω cm^2^^38^. With HBV exposure (MOI 1:100) from the upper chamber or the lower chamber, the amount of exo-virus appearing in the other compartment was detected by qPCR, representing a titer of HBV. Calculated at 24 h and then 48 h after HBV exposure from either side of the chamber, the amount of exo-virus in the other compartment showed no significant differences (Fig. 2D). These results indicate that the trophoblast cells could facilitate HBV endocytosis and exocytosis regardless of cell polarity.

We noticed that the exo-virus titer was at a much lower level than the infection titer, the transcytosis rate was about 3% after 24 h (Fig. 2D). This might be due to that a considerable amount of HBV having been degraded after endocytosis, therefore resulting in less HBV release. Observed under TEM, there were a large number of virus particles in the Swan71 cells after 48 h of HBV infection, either in the cytoplasm (Fig. 2E–E’), or in lysosomes (Fig. 2F–F’). These phenomena suggest that under HBV infection trophoblast cells may use autophagy as a defense mechanism but that HBV might also take advantage of autophagy to achieve exocytosis.

### Screening of S100As co-localized with HBV in the primary trophoblasts and Swan 71 cells

**Fig. 3:**
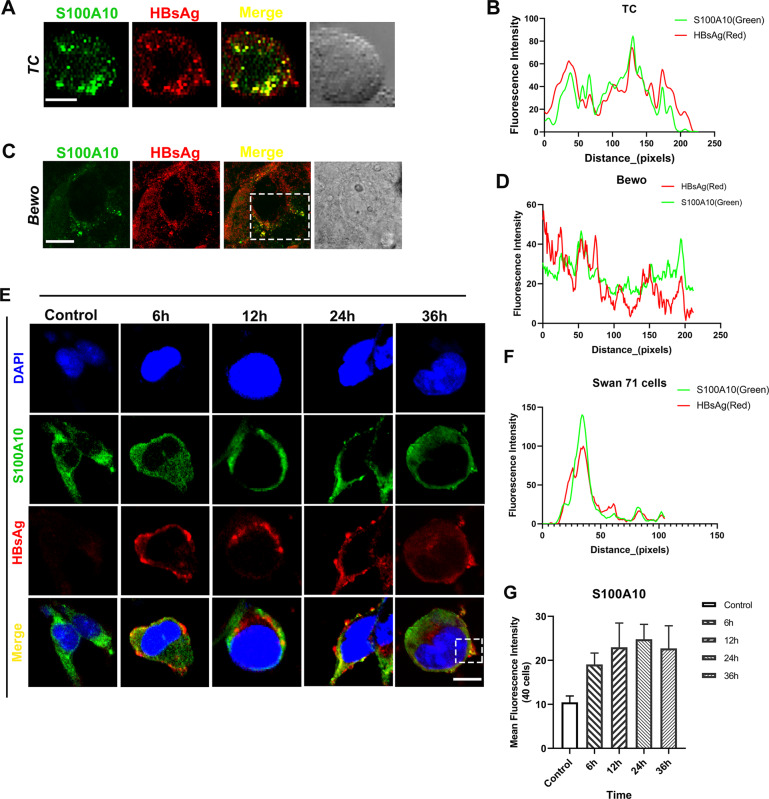
S100A10 is co-localized with HBV in the primary trophoblasts and Swan 71 cells. **A** S100A10 co-localized with HBsAg in trophoblast cell (TC) after being infected with HBV for 24 h. **B** Green and red signal intensity is shown in (**A**). **C** S100A10 was co-localized with HBsAg in BeWo cells after being infected with HBV for 24 h. **D** Green and red signal intensity are shown in (**C**). **E** In the HBV-infected Swan 71 cells, S100A10 co-localized with HBsAg. **F** Green and red signal intensity on the white box shown in (**E**). **G** Fluorescence quantitative analysis of S100A10 shows that there is no obvious relationship between the expression of S100A10 and the duration of HBV infection. Scale bars indicate 10 μm.

To determine whether S100As were related to HBV exocytosis, we performed double immunofluorescent staining of S100As and HBV on primary human trophoblast cells which had been incubated with HBV^+^ serum for 24 h. Results showed that only S100A10 co-localized with HBV, while S100A8, S100 A9, and S100 A12 did not (Fig. 3A, B, Supplementary Fig. 1). These results suggest that S100A10 might directly participate in the intracellular transport and exocytosis of HBV. The co-localization of S100A10 and HBsAg were also observed in HBV-infected BeWo cells after 24 h (Fig. 3C, D) and HBV-infected Swan 71 cells after 6, 12, 24, and 36 h (Fig. 3E, F), indicating the involvement of S100A10 in the exocytosis of HBV. Moreover, by comparing fluorescence intensity, we found that the expression level of S100A10 and the amount of intracellular virus were not significantly related to the durational length of infection (Fig. 3G). This indicates that the rate of HBV exocytosis does not depend on the time of infection.

### Co-localization of S100A10, AnxA2, and HBV in human trophoblast cells and Swan 71 cells

**Fig. 4:**
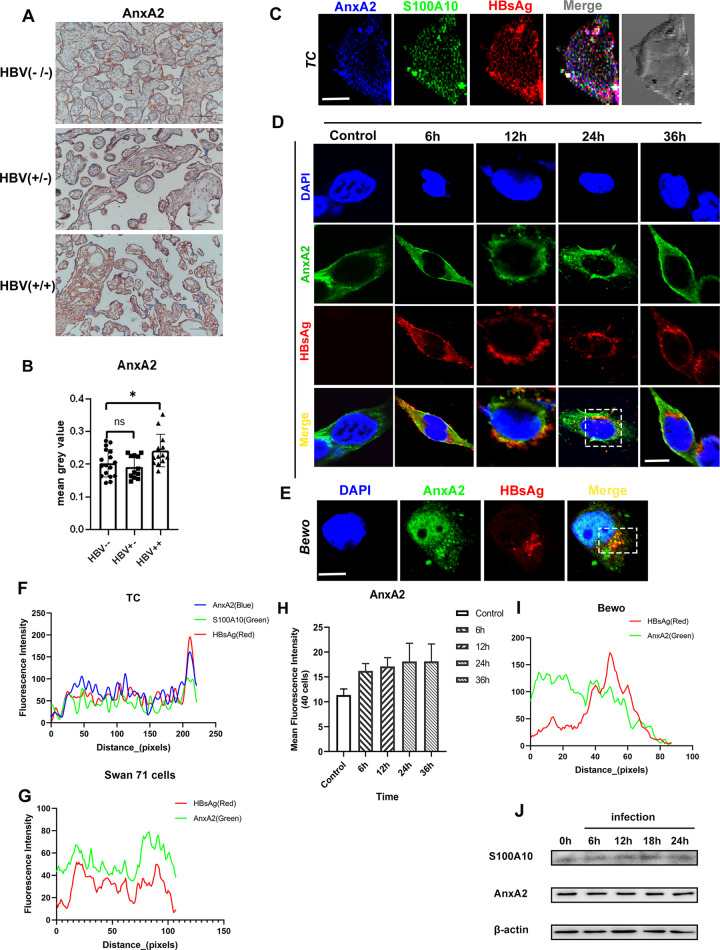
Detection of AnxA2 in HBV−/−, HBV+/−, HBV+/+ placental tissues and co-localization of S100A10, AnxA2, and HBV in human trophoblast cells and Swan 71 cells. **A** Immunohistochemical staining results of AnxA2 in HBV^−/−^, HBV^+/−^, HBV^+/+^ placental tissues. **B** Gray analysis of immunohistochemical staining results of S100As showing that the expression of AnxA2 is only upregulated in HBV^+/+^ placental tissues. **C** Three-color immunofluorescent staining showing that AnxA2 and S100A10 are co-localized with HBV in HBV-infected trophoblast cells. **D** In the HBV-infected Swan 71 cells, AnxA2 was co-localized with HBsAg. **E** AnxA2 and HBsAg were co-localized in the BeWo cells after being infected with HBV for 24 h. **F** Blue, green, and red signal intensity of the white box shown in (**C**). **G** Green and red signal intensity of the white box shown in (**D**). **H** Fluorescence quantitative analysis of AnxA2 showing that there is no obvious relationship between the expression of AnxA2 and the duration of HBV infection. **I** Green and red signal intensity of the white box shown in (**E**). **J** In swan 71 cells infected with HBV, the expression of S100A10 and AnxA2 was increased and the expression level of S100A10 was related to the duration length of infection, while the expression of AnxA2 was not. Scale bars indicate 10 μm.

S100A10 requires the ligand AnxA2 to perform its transport function^39^. Our immunohistochemical staining showed that the expression of AnxA2 was significantly higher in HBV^+/+^ placental tissue, but decreased in HBV^+/−^ placental tissue as compared to controls, (Fig. 4A, B). These results suggest that AnxA2 might incorporate S100A10 in the mechanism of HBV intrauterine transmission. The co-localization of S100A10, AnxA2, and HBsAg was observed in the trophoblast cells and BeWo cells after 24 h exposure to human HBV^+^ serum (Fig. 4C, E, F, I). We then performed double immunofluorescent staining of AnxA2 and HBsAg on Swan71 cells after HBV infection for 6, 12, 24, and 36 h, respectively. The colocalization of AnxA2 and HBV was observed at either early or later stages of infection, indicating that AnxA2 is involved in the process of HBV exocytosis (Fig. 4D, G). By comparing the fluorescence intensity and Western blot, we found that the expression of AnxA2 and the amount of intracellular virus was not significantly related to the duration length of infection (Fig. 4H, J), indicating that the rate of HBV exocytosis does not depend upon the time of infection.

### AnxA2 is involved in the HBV-induced autophagy

**Fig. 5:**
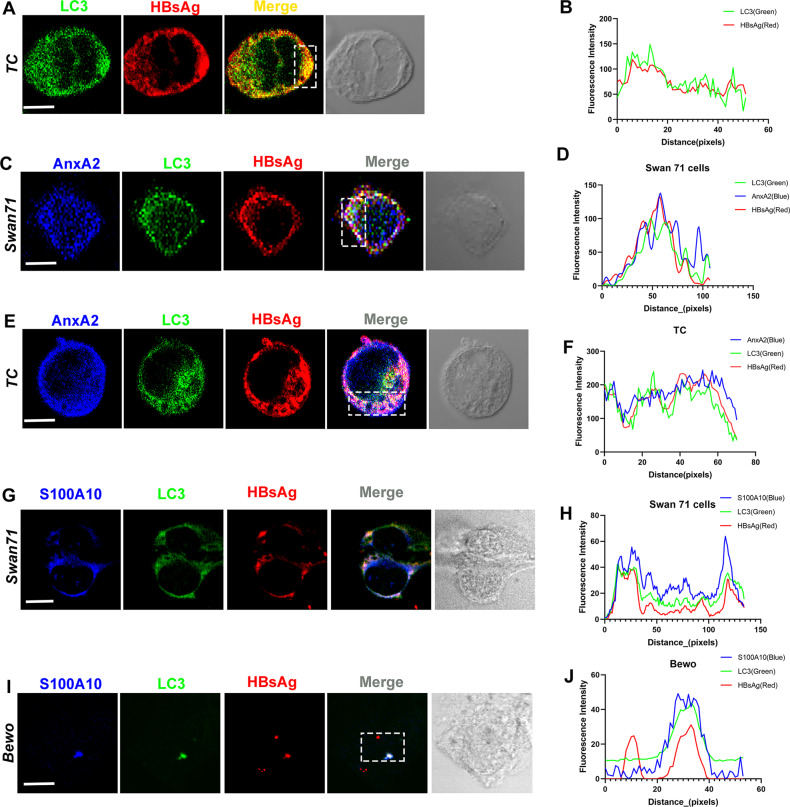
AnxA2 is involved in HBV-induced autophagy. **A** The results of immunofluorescent staining showing that after infection with HBV for 24 h, HBsAg and LC3 co-localized in the trophoblast cells. **B** Red and green signal intensity of the white box shown in (**A**). **C** Three-color immunofluorescent staining showing that after infection with HBV for 24 h, AnxA2, LC3, and HBsAg co-localized in the Swan 71 cells. **D** Blue, green, and red signal intensity of the white box shown in (**C**). **E** Three-color immunofluorescent staining showing that after infection with HBV for 24 h, AnxA2, LC3, and HBsAg co-localized in the trophoblast cells. **F** Blue, green, and red signal intensity of the white box shown in (**E**). **G** In Swan 71 cells infected with HBV for 24 h, S100A10 and LC3 were co-localized with HBsAg. **H** Blue, green, and red signal intensity in (**G**). **I** In BeWo cells infected with HBV for 24 h, S100A10 and LC3 were co-localized with HBsAg. **J** Blue, green, and red signal intensity in (**I**). Scale bars indicate 10 μm.

Autophagy begins with the formation of the phagophore which engulfs cytoplasmic proteins. This LC3, as an autophagosome marker, participates in the early autophagy events and is finally incorporated into autophagosomes^40^. Using double immunofluorescent staining, we observed the co-localization of LC3 and AnxA2 in primary human trophoblasts cells infected with human HBV^+^ serum (Supplementary Fig. 5A). This suggests that LC3 could also serve as a marker of autophagosomes occurring in the process of autophagy in trophoblasts. The HBV-induced autophagy is mediated by HBx protein in liver cells^41^. We observed that LC3 and HBV were co-localized in HBV-infected human trophoblast cells (Fig. 5A, B), indicating that part of HBV is present in autophagosomes. Three-color immunofluorescent staining of the HBV-infected Swan71 cells showed that HBV and AnxA2 would aggregate to the same LC3-presented autophagosomes (Fig. 5C, D). Co-localization of HBV, AnxA2, and LC3 was also observed in human trophoblasts (Fig. 5E, F). Furthermore, the co-localization of HBV, S100A10, and LC3 were observed in both Swan 71 cells and BeWo cells (Fig. 5G–J). These data suggest that HBV and AnxA2, S100A10 could be transported to the same autophagosomes and then into trophoblast cells to complete exocytosis.

### S100A10/AnxA2 regulates HBV exocytosis through MVB-mediated membrane fusion

**Fig. 6:**
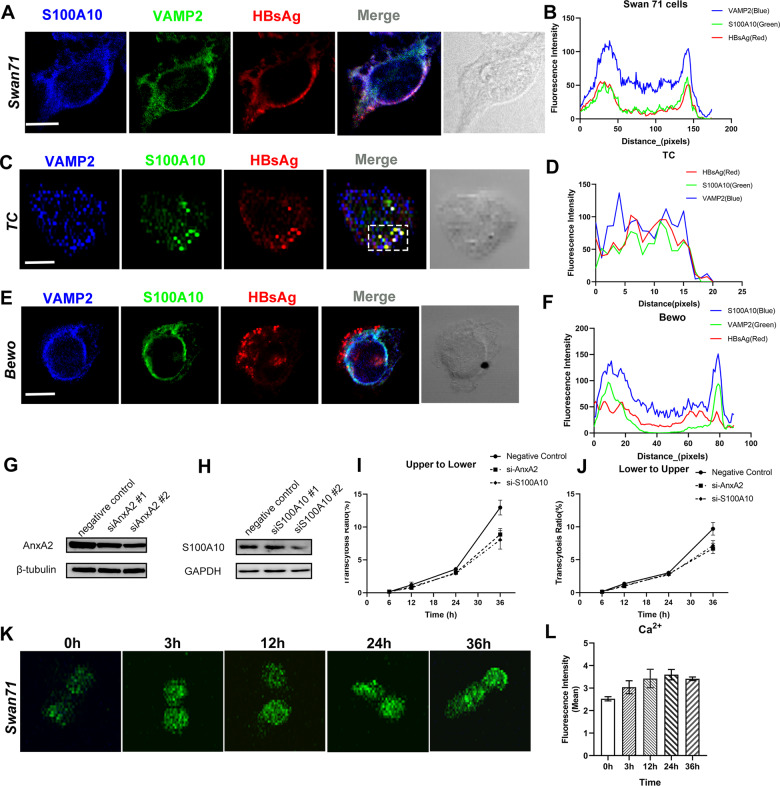
S100A10/AnxA2 regulates HBV exocytosis through MVB-mediated membrane fusion in human trophoblastic cells and Swan 71 cells. **A** Three-color immunofluorescent staining shows that after infection with HBV for 24 h, AnxA2, LC3, and HBsAg co-localized in the Swan 71 cells. **B** Blue, green, and red signal intensity of the white box shown in (**A**). **C** In trophoblast cells infected with HBV for 24 h, VAMP2 and S100A10 co-localized with HBsAg. **D** Blue, green, and red signal intensity of the white box shown in (**C**). **E** In BeWo cells infected with HBV for 24 h, VAMP2 and S100A10 co-localized with HBsAg. **F** Blue, green, and red signal intensity of the white box shown in (**E**). **G** AnxA2 knockdown efficiency was detected with western blotting. β-tubulin was used as the internal control. **H** S100A10 knockdown efficiency was detected with western blotting. β-tubulin was used as the internal control. **I**, **J** HBV-containing serum was added to either upper chamber or lower chamber for infection, and medium from the lower chamber or the upper chamber was collected after the indicated periods of time. Transcytosed HBV-DNA exhibited a slight decrease in AnxA2 or S100A10 knockdown Swan71 after 48 h of infection (lower to upper: AnxA2 siRNA *P* = 0.0197, S100A10 siRNA *P* = 0.0067) (upper to lower: AnxA2 siRNA *P* = 0.002, S100A10 siRNA *P* = 0.0026.), compared with negative control Swan71 cells. **K**, **L** After HBV infection, the Ca2+ concentration in Swan71 cells increased significantly and changed with the increase of infection time. Scale bars indicate 10 μm.

The autophagy process is associated with the formation of MVB^42^. In mammalian cells, MVB fuses with the mature phagosome to form an amphisome, which eventually either fuses with lysosomes to degrade internalized substances or is transported to the plasma membrane to achieve exocytosis^43^. We questioned whether HBV/AnxA2 contained autophagosomes that could fuse with MVB toward exocytosis in trophoblast cells. To test this, we firstly carried out double-color immunofluorescent staining with HBsAg and VAMP2/SNAP25, the components of the membrane fusion complex^44^. VAMP2 and SNAP25 were co-localized with HBsAg (Supplementary Fig. 2A, B and Supplementary Fig. 3A, B), indicating that MVB-mediated membrane fusion had occurred in the trophoblast cells after HBV infection. As we showed earlier in Fig. 2F–F’, MVB contained virus particles that were also observed by TEM in the HBV-infected Swan71 cells. We performed three-color immunofluorescent staining with S100A10, VAMP2, and HBsAg. The co-localization of these three molecules was observed in both trophoblast cells, Swan71 cells, and BeWo cells (Fig. 6A–F). These results strongly suggest that the S100A10/AnxA2 complex and HBV-contained autophagosomes fuse with MVB toward exocytosis in trophoblast cells. We silenced either AnxA2 or S100A10 with specific siRNA in trophoblast cells (Fig. 6G, H). The quantification is shown in Supplementary Fig. 4A–D. The results of subsequent Transwell experiments showed that the amount of exo-virus was reduced in both directions, while the control group showed no effect (Fig. 6I, J).

During exocytosis, membrane fusion and cytoskeleton rearrangement are accompanied by Ca^2+^ activities^45^, we examined intracellular Ca^2+^ signals using a calcium fluorescence probe. Observed at 0, 3, 12, 24, and 36 h after HBV infection in Swan71 cells, the intracellular Ca^2+^ concentrations of Swan71 increased significantly with HBV infection time (Fig. 6K, L). These results indicate that HBV and S100A10/AnxA2-contained transport vesicles eventually formed membrane fusion complexes to induce cytoskeleton rearrangement and exocytosis.

### A proportion of HBV hijacks the autophagic protein secretion pathway for exocytosis

**Fig. 7:**
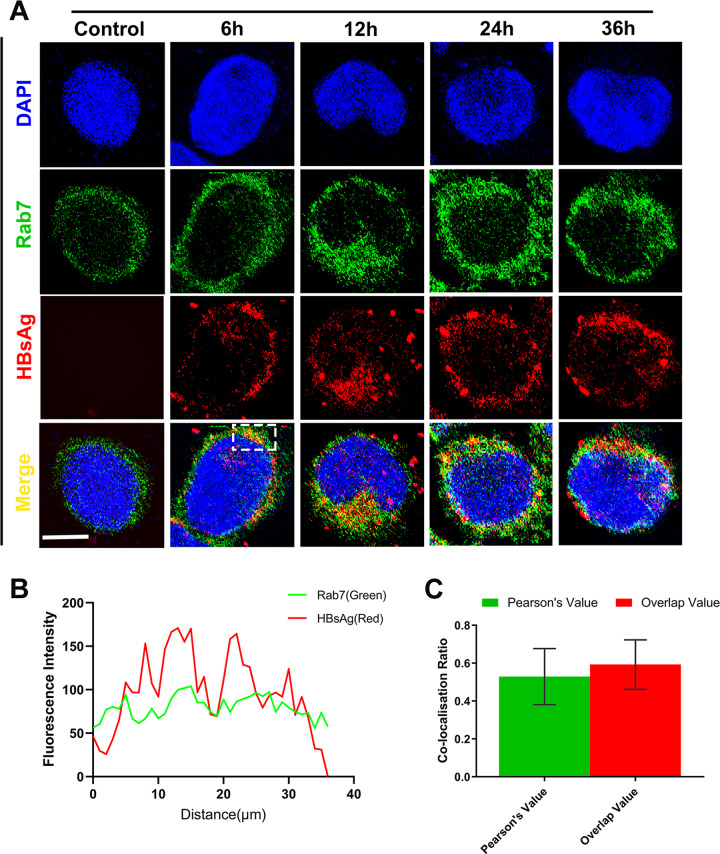
Rab7 mediates HBV entry into the lysosome. **A** Co-localization of Rab7 and HBsAg in Swan 71 cells infected with HBV after 6, 12, 24, and 36 h. **B** Green and red signal intensity of the white box shown in (**A**)**. C** Pearson index and overlap index of fluorescence in 40 cells. Scale bars indicate 10 μm.

In the HBV-infected Swan71 cells, the exo-virus number was relatively small. A similar result was noted from our Transwell experiments. As one of the cell defense mechanisms, pathogenic microorganisms in the cytoplasm could be degraded through autophagy^34^. In HBV-infected hepatic cells, Rab7 interacts with LC3 and PI3P to direct the microtubule plus-end transportation of autophagic vesicles, resulting in the fusing of mature autophagosomes and lysosomes for content degradation^33^. We performed double immunofluorescent staining with HBsAg and Rab7. As shown in Fig. 7A, HBsAg and Rab7 had significant co-localization. Observation of Swan71 cells infected with HBV for 48 h by TEM also revealed a large number of dense lysosomes, many of which were encapsulated with virus particles (Fig. 2F–F’). Fluorescence intensity quantification showed that the co-localization signal of HBV and Rab7 in the early stage of infection was relatively strong (Fig. 7B, C). This may be related to defense response. No obvious changes in overlapping fluorescence intensity were observed, indicating that the cells may have turned into a relatively healthy condition in the later period after HBV exocytosis. These results suggest that in HBV-infected trophoblast cells, the majority of the endocytosed virus may be transported to the lysosome with the assistance of Rab7 for degradation, but that a small proportion would evade such degradation and undergo exocytosis instead.

## Discussion

Hepatitis B virus is a hepatotropic virus that can establish a long-lasting chronic infection in humans. Hepatitis B virus infection has been a major global public health problem. Although effective vaccines have been developed, there are still more than 257 million people infected with hepatitis B virus worldwide, and more than 887,000 people die from liver failure or hepatocellular carcinomas caused by chronic hepatitis B virus infection year^46^. People infected with the hepatitis B virus have a 30-fold increase in the risk of hepatocellular carcinoma, and more than 50% of hepatocellular carcinomas worldwide are caused by chronic hepatitis B virus infection^47^.

A number of studies have reported, as we do here, that MTCT-HBV infection could occur through intrauterine transmission^10^. Attempts to completely block MTCT-HBV infection have represented a long clinical challenge due to the lack of understanding of its underlying mechanisms. In a previous report, Liu drawn the venous blood of 144 HBV-infected mothers and their newborns, tested HBV-DNA, HBV antibodies, and AST/ALT. The results showed that mothers with HBeAg (+) or HBV DNA (+) are more likely to infect fetuses, with an infection rate of 70.5% and 61.1%. The average value of AST and ALT of infected fetuses is (61.2 ± 31.3) IU, (24.7 ± 14.9) IU, which is significantly higher than uninfected fetuses^48^. However, in our practice clinic observations, so far, we have not detected such differences. In this study, we discovered that HBV could infect trophoblast cells, and then, during the autophagy-mediated defense response to kill part of the virus, a small part of HBV could hijack the components of the autophagy process to achieve exocytosis. This may provide the key revelationas to how the mechanisms of HBV intrauterine transmission occur. Regarding the HBV transcytosis experiments using trophoblast cell lines, we noticed that BeWo cells clumped together and did not form a monolayer whereas Swan 71 cells could be easily seeded and readily grew into a monolayer. This might be due to the characteristics of these different cell lines. However, considering our observations in immunofluorescence staining experiments, we found that the morphology and quantity of HBV-MVBs colocalization in BeWo cells were similar to those in Swan 71 cells and in primary trophoblast cells. We, therefore, considered the Swan 71 cell line as sufficient to illustrate the efficiency of HBV exocytosis.

The autophagy pathway forms vesicles to encapsulate the virus particles or virus components in the cytoplasm and transport them to lysosomes to degrade them, thereby promoting the elimination of intracellular infections^49^. In Sindbis virus (SIN)-infected mice, Beclin1 reduces intracellular viral titers by enhancing the heterologous phagocytosis of SIN and protects the mice from fatal encephalitis^50^. In our study, we observed a large number of HBV vesicles co-localized with Rab7 in infected Swan 71 cells. In hepatic cells, HBV could activate Rab7 to promote the conversion of MVB and autophagosomes to lysosomes, resulting in reduced virus production^33^. Due to a weakened immune response, the viral load is greatly increased in the early stages of chronic infection^51^. It is likely that the autophagy-lysosomal pathway mediated by Rab7 can reduce the secretion of HBV to a certain extent, thereby avoiding a severe immune response leading to acute hepatitis. The activation of Rab7 may be one of the strategies of the virus to reduce immune responses.

Some viruses have evolved various mechanisms to counter the host’s antiviral effects by evading or even using the host’s autophagy processes to promote their own replication and pathogenic processes^52^. For instance, Herpes viruses, such as Kaposi’s sarcoma-associated herpesvirus, Symes herpes virus, and molluscum contagiosum infection, all can prevent Atg3 mediation by expressing the viral FLICE-like inhibitory protein-guided LC3 coupling step, thereby inhibiting autophagy^53^. HBV may enhance its own replication by inducing autophagy in cultured cells^54^. The inhibition of autophagy reduces the expression of the HBX gene and affects the nuclear localization of the HBV core protein^54^. In this way, autophagy seems to play a double-edged sword in the infection process of pathogenic microorganisms.

In the serum of hepatitis B patients, cytokines such as TNF-α and interferon γ can upregulate transcription factor C/EBPβ, which then elevate the expression of S100A8/A9^55,56^. The high expression of S100A8/A9 disrupts the integrity of vascular endothelial cells, affecting cell permeability^57^ and causing apoptosis in various cells^58–60^. Therefore, HBV infection mediated inflammation might induce a high expression of S100A8/A9/A12 in the placenta, resulting in damage to the placental barrier and high risk of HBV-MTCT transmission via blood-placental means. In the present study, though we found that the expressions of S100A8/A9/A10/A12 were increased in the placenta of HBV-positive patients, only S100A10 was related to the exocytosis of HBV in trophoblasts.

AnxA2 is a calcium-dependent phospholipid binding protein, which exists as a monomer and as a heterotetrametric complex together with S100A10^21^. AnxA2 and the AnxA2/S100A10 complex have been related to many processes, such as endocytosis, exocytosis, and membrane organization^17,18^. AnxA2 and A2t are involved in the pathogenic process of many viruses. It has been suggested that at least 13 viruses including HCV, HPV, and HIV-1 are associated with AnxA2 or A2t during binding, endocytosis, and egress^61^. Our study shows that A2t is involved in the process of HBV exocytosis in trophoblasts. In this, AnxA2 was transferred from the cytoplasm to the surface of autophagosomes, contained HBV and recruited S100A10 to form the S100A10–AnxA2 complex. The autophagosome then fused with MVB to form the amphisome in a Rab11-dependent process. The S100A10–AnxA2 complex then recruited VAMP2, SNAP25 for membrane fusion. Through activating calcium channels and the PI3K/FAK pathway, the PI3K/FAK pathway was induced to stimulate Ca^2+^ activity and MF/MT-dependent cytoskeleton rearrangement where HBV eventually emerged via exocytosis. Another part of the autophagosome containing HBV was degraded by fusion with lysosomes under the action of Rab7. This process might reduce the amount of HBV virus in the cell and protected the cell from fatal and acute inflammation.

In conclusion, this study shows that an unconventional protein secretion pathway that depends on autophagy may be hijacked by HBV to complete the process of intracellular transport and exocytosis. In the HBV infected trophoblasts, AnxA2–S100A10 complex-mediated exocytosis could result in HBV intrauterine transmission. This may also provide some insights for understanding the viral exocytosis pathways and intracellular transport.

## Supplementary information

Supplementary Table

Supplementary Figures

## Data Availability

All data generated during this study are available within the article.
