# Supplementary material for: The S100A10–AnxA2 complex is associated with the exocytosis of hepatitis B virus in intrauterine infection
Source: Lab Invest. 2021 Oct 13;102(1):57–68. doi: 10.1038/s41374-021-00681-8 (PMC8512653; doi:10.1038/s41374-021-00681-8)
Supplement: Supplementary file 1 — Supplementary Table [file 41374_2021_681_MOESM1_ESM.pdf]

Table 1. Key Materials and resources

REAGENT or RESOURCE	SOURCE	IDENTIFIER
Antibodies		
Rabbit anti-AnxA2	Abcam	Cat. #: ab41803
Rabbit anti-S100A10	Abcam	Cat. #: ab76472
Mouse anti-S100A10	Santa Cruz	Cat. #: sc-81153
Rabbit anti-VAMP2	Abcam	Cat. #: ab181869
Mouse anti-LC3B	Abcam	Cat. #: ab243506
Rabbit anti-CK7	Abcam	Cat. #: ab181598
Rabbit anti-Rab7	Abcam	Cat. #: ab126712
Goat anti-HBsAg	Shanghai Boyao	N/A
Mouse anti-S100A9	Santa Cruz	Cat. #: sc-376772
Mouse anti-AnxA6	Santa Cruz	Cat. #: sc-365582
Mouse anti-S100A12	Santa Cruz	Cat. #: sc-101347
Mouse anti-S100A11	Santa Cruz	Cat. #: sc-390250
Mouse anti-S100A8	Santa Cruz	Cat. #: sc-48352
Mouse anti-α-tubulin (clone 1E4C11)	Proteintech	Cat. #: 66031-1-Ig
Mouse anti-β-actin	Santa Cruz	Cat. #: sc-69879
Goat anti-mouse IgG (H&L) [HRP]	Genscript	Cat. #: A00160
Goat anti-rabbit IgG (H&L) [HRP]	Genscript	Cat. #: A0098
Alexa Fluor 488 donkey anti-mouse IgG	Thermo Fisher Scientific	Cat. #: A21202
Alexa Fluor Plus 594 donkey anti-goat IgG	Thermo Fisher Scientific	Cat. #: A32758
Alexa Fluor Plus 488 donkey anti-rabbit IgG	Thermo fisher Scientific	Cat. #: A32790
Alexa Fluor 405 donkey anti-rabbit IgG	Abcam	Cat. #: ab175649
**Bacterial and Virus Strains**		
Hepatitis B virus	Women's Hospital of Zhejiang University	N/A
**Biological Samples**		
Human placental tissues	Women's Hospital of Zhejiang University	N/A
Human primary placental trophoblasts	Women's Hospital of Zhejiang University	N/A
Human HBV-containing serum	Women's Hospital of Zhejiang University	N/A
**Chemicals, Peptides, and Recombinant Proteins**		
Collagenase I	Yeason	Cat. #: 40507ES60
D-PBS	Beyotime	Cat. #: C0221G
Trypsin-EDTA	Yeason	Cat. #: 40127ES60
DAPI	Solarbio	Cat. #: C0065
RIPA buffer	Solarbio	Cat. #: R0010

Fluor-4-AM	Beyotime	Cat. #: S1060
Critical Commercial Assays		
Viral DNA Kit	Omegabiotek	Cat. #: D3892
BCA Protein Assay Kit	Thermo Fisher Scientific	Cat. #: 23225
Oligonucleotides		
HBV precore-specific primer	Bhat et al., 2007	N/A
Recombinant DNA		
HBV 1.3-mer WT replicon	Addgene	Cat. #: plasmid 65459
