# Supplementary material for: The S100A10–AnxA2 complex is associated with the exocytosis of hepatitis B virus in intrauterine infection
Source: Lab Invest. 2021 Oct 13;102(1):57–68. doi: 10.1038/s41374-021-00681-8 (PMC8512653; doi:10.1038/s41374-021-00681-8)

Supplementary Figure 1

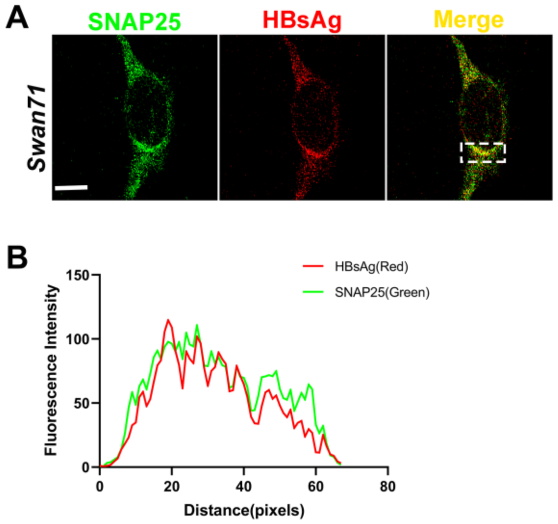

Supplementary Figure 2

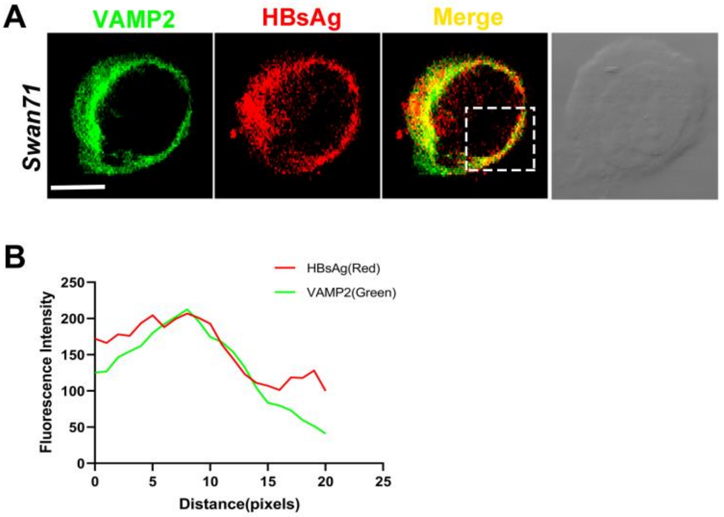

Supplementary Figure 3

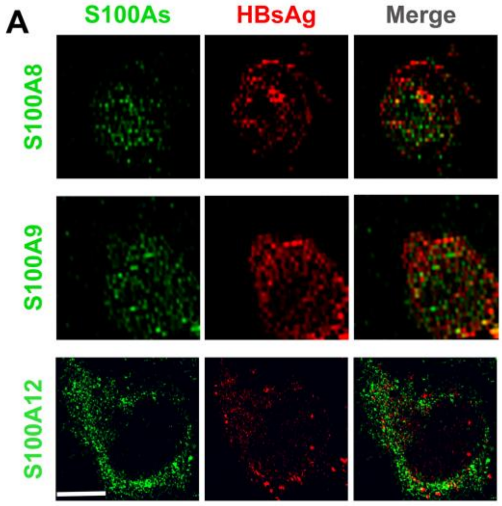

Supplementary Figure 4

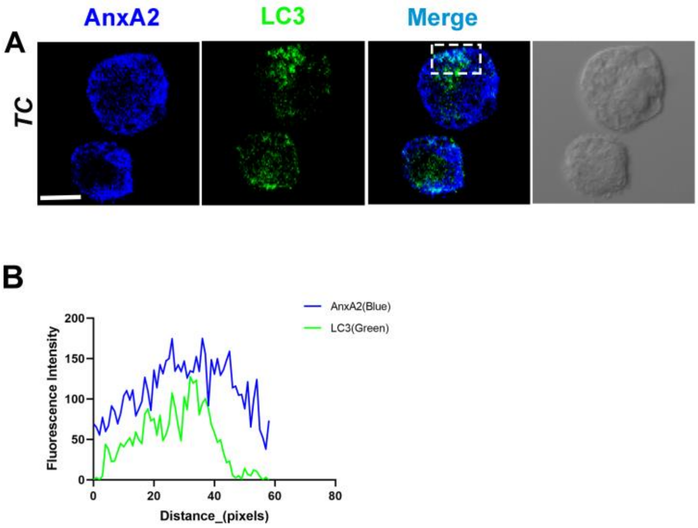

Supplementary Figure 5

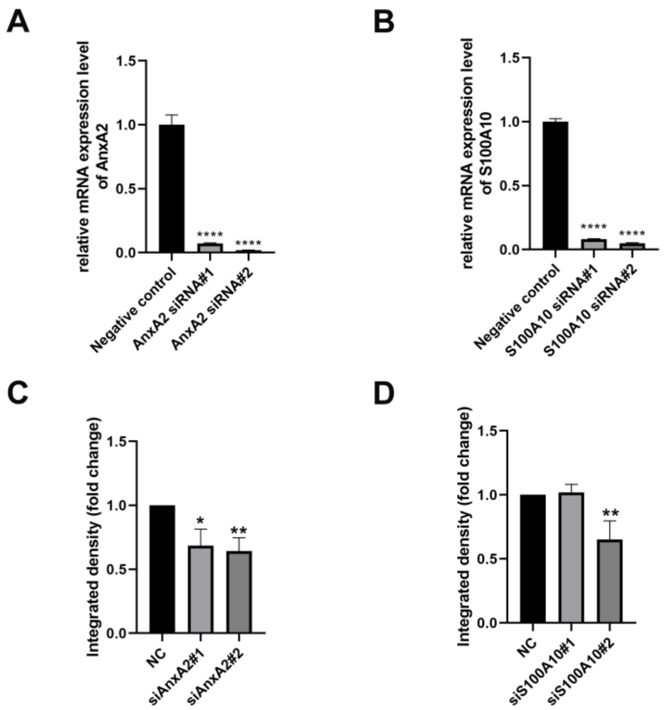

Supplement: Supplementary file 2 — Supplementary Figures [file 41374_2021_681_MOESM2_ESM.pdf]
